# Oral Streptococci Biofilm Formation on Different Implant Surface Topographies

**DOI:** 10.1155/2015/159625

**Published:** 2015-07-26

**Authors:** Pedro Paulo Cardoso Pita, José Augusto Rodrigues, Claudia Ota-Tsuzuki, Tatiane Ferreira Miato, Elton G. Zenobio, Gabriela Giro, Luciene C. Figueiredo, Cristiane Gonçalves, Sergio A. Gehrke, Alessandra Cassoni, Jamil Awad Shibli

**Affiliations:** ^1^Department of Periodontology and Oral Implantology, Dental Research Division, Guarulhos University, Praça Tereza Cristina 229, 07023-070 Guarulhos, SP, Brazil; ^2^Department of Operative Dentistry, Dental Research Division, Guarulhos University, Praça Tereza Cristina 229, 07023-070 Guarulhos, SP, Brazil; ^3^Department of Oral Implantology, PUC Minas, Avenida Dom Cabral 500, Prédio 46, Coração Eucarístico, 30535-901 Belo Horizonte, MG, Brazil; ^4^Biotecnos-Tecnologia e Ciência Ltda, Rua Dr. Bozano, 571 Centro, 97015-001 Santa Maria, RS, Brazil

## Abstract

The establishment of the subgingival microbiota is dependent on successive colonization of the implant surface by bacterial species. Different implant surface topographies could influence the bacterial adsorption and therefore jeopardize the implant survival. This study evaluated the biofilm formation capacity of five oral streptococci species on two titanium surface topographies. *In vitro* biofilm formation was induced on 30 titanium discs divided in two groups: sandblasted acid-etched (SAE- *n* = 15) and as-machined (M- *n* = 15) surface. The specimens were immersed in sterilized whole human unstimulated saliva and then in fresh bacterial culture with five oral streptococci species:* Streptococcus sanguinis*,* Streptococcus salivarius*,* Streptococcus mutans*,* Streptococcus sobrinus*, and* Streptococcus cricetus*. The specimens were fixed and stained and the adsorbed dye was measured. Surface characterization was performed by atomic force and scanning electron microscopy. Surface and microbiologic data were analyzed by Student's *t*-test and two-way ANOVA, respectively (*P* < 0.05).* S. cricetus*,* S. mutans,* and* S. sobrinus* exhibited higher biofilm formation and no differences were observed between surfaces analyzed within each species (*P* > 0.05).* S. sanguinis* exhibited similar behavior to form biofilm on both implant surface topographies, while* S. salivarius* showed the lowest ability to form biofilm. It was concluded that biofilm formation on titanium surfaces depends on surface topography and species involved.

## 1. Introduction

Establishment of the dental subgingival microbiota is dependent on successive colonization of the tooth surface by several bacterial species [1]. Each of these bacterial species appears to facilitate enamel surface colonization by the next wave of bacterial settlers, resulting in the establishment of an anaerobic Gram-negative microbiota [2]. So far, it is believed that a similar pattern of the same colonization process may occur on the titanium implant surfaces [3–5].

However, the different implant surface topographies could influence the bacterial adsorption [6–8]. Physical and chemical factors may affect the attachment of biofilms to hard surfaces. The surface roughness at micrometer level can increase the surface area and hence increase the bacterial colonization. Roughness also provides protection from shear forces and increases the difficulty of cleaning methods. Furthermore, Kolenbrander et al. [2] have shown that supragingival plaque formation, after initial bacterial colonization, was faster on a rough surface. The roughness of different dental implant surfaces can work like grooves for initial periodontal pathogen adhesion [6, 9, 10].

The oral streptococci are members of the indigenous microbiota mainly in the supragingival environment [11] and species of mutans group such as* Streptococcus mutans, Streptococcus sobrinus, *and* Streptococcus cricetus* were related to individuals with teeth because they are able to adhere to nonshedding surfaces, with* S. mutans* being the most prevalent species in humans [12]. Other species such as* Streptococcus sanguinis *and* Streptococcus salivarius *are commonly found in healthy periodontal individuals and the latter is related to mucosal surfaces; besides it can contribute to the coaggregation of pathogenic bacteria, such as* Porphyromonas gingivalis*. Thus the oral streptococci are considered the pioneer colonizers and might participate in the process, which can lead to implant failure on the long term [6].

Therefore, the aim of this* in vitro* study was to verify the ability of five oral streptococci species to form biofilm on two different titanium surface topographies.

## 2. Material and Methods

### 2.1. Implant Surface Topography

Thirty discs (5 mm diameter and 3 mm thickness) made of grade-4 titanium (Implacil De Bortoli, Sao Paulo, SP, Brazil) were prepared with 2 surface topographies: as-machined (M) and sandblasted acid-etched (SAE) surfaces. The titanium discs with sandblasted acid-etched surface were blasted with 50–100 *μ*m TiO_2_ particles. After sandblasting, the specimens were ultrasonically cleaned with an alkaline solution, washed in distilled water, and pickled with maleic acid.

### 2.2. Implant Surface Characteristics

The samples were first checked for chemical composition with XPS/ESCA (X-ray photoelectron spectroscopy/electron spectroscopy for chemical analysis), and no significant pollution was detected [13]. The topographies at the microscale were then visualized using routine scanning electron microscopy (SEM) control. At the nanoscale, the SEM confirmed that both surface types were nanosmooth, following the current definition [13, 14]. The sole difference between these 2 tested implant types was therefore the specific surface microtopography.

Atomic force microscopy (AFM, PicoSPM I plus 2100 PicoScan Controller, in contact mode) was used for the surface topography analysis, in contact mode. The AFM scanned areas of 60 *μ*m × 60 *μ*m of each specimen. The measured parameters, such as the arithmetic average of all profile point absolute values (Ra), the root-mean-square of all point values (Rq), and the average absolute height values of the five highest peaks and the depths of the five deepest valleys (Rz), were measured for each group. Representative images of the surfaces of each group of specimens were also taken by scanning electronic microscopy.

### 2.3. Strains

*Streptococcus sanguinis *(ATCC 10556),* Streptococcus salivarius* (ATCC 7073),* Streptococcus mutans *(ATCC 25175) and* Streptococcus sobrinus* (ATCC 33478), and* Streptococcus cricetus* (ATCC 19642) were used in this study in biofilm formation.

### 2.4. Saliva Coating of the Specimens

Unstimulated saliva from 6 healthy nonsmoker and systemic healthy donors was collected for one hour per day, for seven days. Then the saliva samples were sterilized and frozen at −20°C until a total of 500 mL was collected per donor. All donors signed the informed consent. Subsequently, the saliva samples were pooled and centrifuged (30 min; 4°C; 27,000 ×g). The supernatant was pasteurized (60°C, 30 min) to inactivate endogenous enzymes, recentrifuged (30 min, 4°C; 27,000 ×g) in sterile bottles, and stored at −20°C. The pasteurization efficacy was evaluated by plating 100 *μ*L of saliva onto brain heart infusion (BHI) agar and by observing the absence of bacterial growth after 72 hours. The sterile disks were placed in a sterile 24-well polystyrene cell culture plate containing 500 *μ*L of saliva for 4 hours to allow salivary pellicle formation.

### 2.5. Biofilm Formation Assay

After coating period, saliva was aspirated from each well and replaced with 500 *μ*L of BHI broth (double concentrated) and 500 *μ*L of saliva. Inocula were prepared by harvesting each standard reference strain cell from BHI agar plates previously inoculated and incubated under microaerophilic conditions for 24 hours (candle jar, 37°C). The bacterial cells were suspended in sterile saline solution, adjusting the turbidity to OD_630_0.15 (~10^6^ UFC/mL). Each well was inoculated with 100 *μ*L of this* inoculum *suspension. Plates were then incubated for 16 hours under microaerophilic conditions. Afterwards, the specimens were gently washed in sterile saline solution three times in order to remove unattached cells.

The specimens with remaining attached bacteria were fixed using 0.25 mL of 2.5% glutaraldehyde per well for 15 min and, subsequently, air-dried. The specimens were transferred to clean well plates and were stained with 0.25 mL of crystal violet for 5 min. Excess stain was rinsed off by placing the microplate under running tap water, and after this it was air-dried. The specimens were transferred to clean tubes, and, in order to resolubilize the dye bound to the adherent cells on specimen surfaces, 0.3 mL of ethanol was added per well. The supernatant was transferred to a clean 96-well microplate, and the absorbance was measured at 570 nm using an automated 96-well microplate reader.

### 2.6. Statistical Analysis

The surface characterization was tested using Student's *t*-test. Two-way analysis of variance (ANOVA) was used in order to compare the groups of species within the same group of implant surface topography and to verify possible differences among specimen surfaces within the same species (*α* = 0.05).

## 3. Results

### 3.1. Surface Characterization of Implants Surfaces Substrata

**Figure 1:**
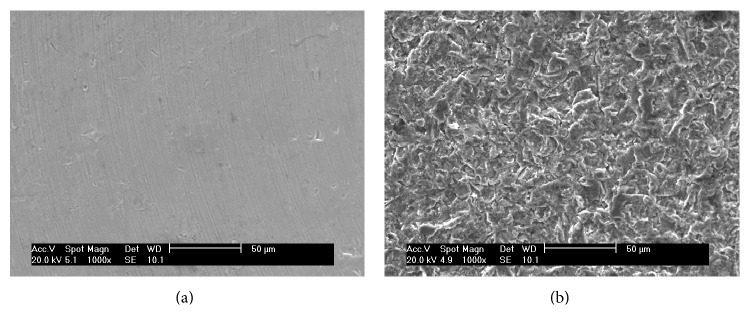
Scanning electron microphotograph of the implant surface topography: (a) as-machined implant surface and (b) sandblasted acid-etched surface.

Scanning electronic microscopy showed that M group exhibited only the grid of machining (Figure 1(a)). On the other hand, the SAE exhibited peaks and valleys with diverse irregularities (Figure 1(b)).

**Figure 2:**
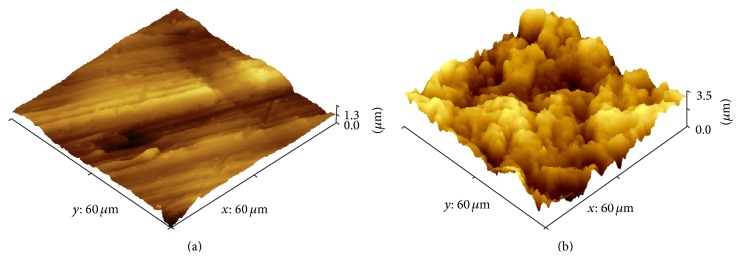
Atomic force microscopy (AFM) of the implant surface topography: (a) as-machined implant surface and (b) sandblasted acid-etched surface.

The surfaces were characterized by atomic force microscopy, which revealed differences between the surfaces (*P* < 0.0001). M showed only the machining grids with peaks of 1.3 *μ*m and some regions that were almost flat (Figure 2(a)). The SAE exhibited irregular surfaces with peaks of about 6.5 *μ*m (Figure 2(b)). The roughness values are shown in Table 1.

### 3.2. *In Vitro* Determination of Microbial Adhesion

**Figure 3:**
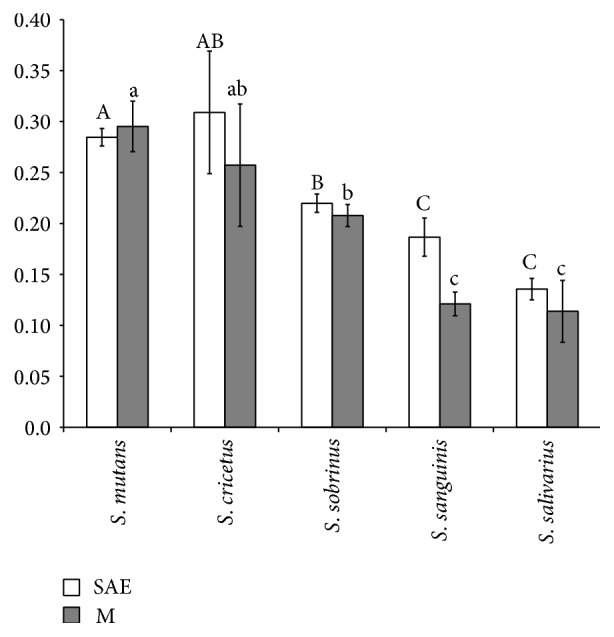
Mean ± standard deviation of the amount of adsorbed dye released after the assay (*P* > 0.05; two-way ANOVA). Letters: differences among biofilm accumulated by each species (*P* < 0.05; two-way ANOVA/Tukey test). Different letters indicate groups with distinct characteristics. Capital letters compare SAE surfaces, while lower case letters compare M surfaces.

The biofilm forming ability was evaluated and the means of readings are shown in Figure 3. The group mutans streptococci (*S. cricetus, S. mutans,* and* S. sobrinus*) exhibited higher levels of biofilm formation and no differences were observed between surfaces analyzed within each species (*P* > 0.05). It was observed that although* S. cricetus *exhibited the highest ability to form biofilm on SAE, among all species, within this species this difference was not significant (*P* > 0.05) between the surfaces analyzed.

**Figure 4:**
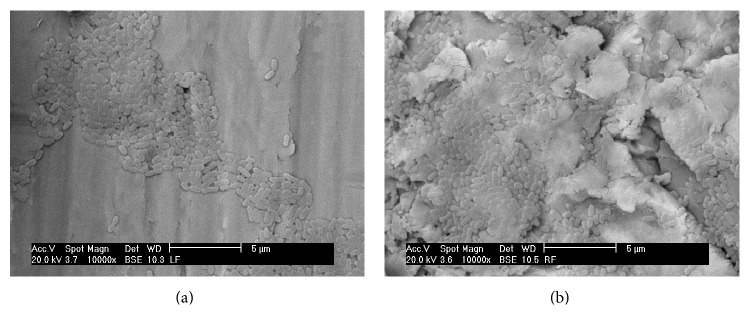
Representative scanning electron microscopy (×10,000) in a back scattering mode (BSE) of the* Streptococcus sanguinis* in (a) as-machined (M) and (b) sandblasted acid-etched (SAE) surface. Note proliferation of the* S. sanguinis* in the pitches and notches of the SAE surface.

*S. sanguinis *exhibited a similar behavior to form biofilm on both implant surface topographies (Figure 4), and their ability to do so was lower than that of the group mutans streptococci species. The lowest ability was observed for* S. salivarius*.

## 4. Discussion

Titanium has been widely used as a component of dental implants since the 1970s. More than the improvements in biomechanical performance, these modifications on implant surfaces lead to other biological responses, such as differences in the protein adsorption profiles [15, 16], attachment, cell proliferation and differentiation, and fibrin adhesion [17]. The present study presented the biofilm forming ability of 5 oral streptococci species on two different types of surfaces.

The blasting process with titanium oxide particle (50–100 *μ*m) and maleic acid solution etching modified substantially the surface, which was indeed confirmed by SEM and AFM. AFM revealed higher density of irregularities on R surface as well higher peaks. An earlier study [17] detected different profiles of plasma adsorption depending on surface treatment (acid etching only and blasting plus acid etching processes) and attributed this difference mainly to the changes in physical properties, since minor alterations in chemical composition were detected. On the other hand, Li et al. [18] found differences on titanium surfaces after application of different treatments, including chemical changes such as an oxide layer and surface contamination, and these might exert some influence on biocompatibility issues. Recently, it has been shown that nanosurfaces could impair bacterial adsorption, suggesting that further studies must be done to evaluate the role of implant surface topography on bacterial colonization [19].

However, there is an unclear debate about the link between bacterial contamination and peri-implantitis [20, 21]. These papers suggested that peri-implantitis is pathology of bone-to-implant interface and that bacterial contamination is only the associated consequence, not the triggering factor. However, we must point out that, until now, there are now clear and consistent evidences to follow this idea.

In addition, these surface changes might also influence biofilm formation, since the earlier steps of this process are related to contact surface extension, surface free energy, topography, wettability, hydrophobicity, and other surface traits [17, 18, 22–26].

The results of the present study revealed differences as regards the biofilm forming ability among* S. salivarius, S. cricetus, S. mutans, S. sobrinus, *and* S. sanguinis*. Among them,* S. salivarius *and* S. sanguinis *exhibited the lowest capacity to form biofilm. Two aspects of biofilm forming ability must be pointed out: the specific traits of each species and surface topographies.

Differences on adhesion to glass surface among mutans streptococci group were already observed [27]. The authors found that* S. rattus* adhered less than the other species (*S. sobrinus, S. mutans, *and* S. cricettus*) and attributed these results to different properties of the* S. rattus* surface like negative zeta-potentials. In the present study, three species of mutans streptococci group (*S. mutans, S. cricetus, *and* S. sobrinus*) were evaluated and although the raw values showed a high capacity of* S. mutans *to accumulate biofilm on titanium surface followed by* S. cricetus *and* S. sobrinus*, statistical differences were observed only between* S. mutans *and* S. sobrinus* (*P* < 0.05).

Although roughness seems to promote an increase in the amount of plaque, the biofilm composition did not show substantial changes and the establishment of irreversible attachment in the surface irregularities, where microorganisms are protected against mechanical shear [10]. Despite this, the results of our study demonstrated that biofilm formation does not increase markedly on rougher surfaces.

Oral strains, most of them having high surface free energy, might adhere better to hydrophilic substrata [28]. Differences with regard to surface hydrophobicity could be attributed to the acid etching, which could introduce ^−^OH groups on the surface, thus modifying its chemical properties [29]. According to this hypothesis, these treatments can originate different surfaces, and, consequently, new patterns of adsorbed substances will be originated, which may offer different profiles of receptors for bacterial colonization.

Another issue concerns virulence traits of each species like tooth colonization mechanisms;* S. mutans *apparently attach by adhesin and glucan mediated mechanisms, whereas* S. sobrinus *utilize primarily the latter process [30].

## 5. Conclusions

In conclusion, within the limitations of the study, the present findings showed the following: (a) biofilm formation by oral streptococci might vary according to the species; (b)* S. salivarius *and* S. sanguinis* showed the lowest ability to accumulate biofilm; (c) group mutans streptococci accumulated higher amounts of biofilm; (d) the substratum roughness is not the only issue to be considered with regard to bacterial biofilm formation.

## Figures and Tables

**Table 1:** Mean ± standard deviation of the as-machined (MS) and titanium discs blasted with titanium oxide particles and washed with maleic acid solution (SAE) profilometry.

Implant surface topography^*^	Ra (*μ*m)	Rq (*μ*m)	Rz (*μ*m)
As-machined (M)	0.14 ± 0.02	0.16 ± 0.01	1.61 ± 0.10

Sandblasted acid-etched surface (SAE)	0.87 ± 0.14	1.12 ± 0.18	5.14 ± 0.69

^*∗*^Statistically significant between the implant surface topographies (Student's *t*-test *P* = 0.0001), M < SAE; Ra: arithmetic average of the absolute values of all profile points; Rq: the root-mean-square of the values of all points; Rz: the average value of the absolute heights of the five highest peaks and the depths of the five deepest valleys.
